# The Effect of Hemithyroidectomy in Papillary Thyroid Carcinoma with an Exclusive Involvement of the Recurrent Laryngeal Nerve: A Retrospective Study with a Propensity Score-Matched Analysis

**DOI:** 10.3390/curroncol31060265

**Published:** 2024-06-20

**Authors:** Feng Zhu, Yibin Shen, Lixian Zhu, Linghui Chen, Fuqiang Li, Xiaojun Xie, Yijun Wu

**Affiliations:** The Department of Thyroid Surgery, School of Medicine, The First Affiliated Hospital, Zhejiang University, Hangzhou 310003, China; lwsjn@zju.edu.cn (F.Z.); ander_syb@aliyun.com (Y.S.); 10918188@zju.edu.cn (L.Z.); chenlinghui@zju.edu.cn (L.C.); fqiangli@126.com (F.L.); xiaoj_xie@126.com (X.X.)

**Keywords:** papillary thyroid carcinoma, recurrent laryngeal nerve invasion, hemithyroidectomy, total thyroidectomy, prognosis

## Abstract

Background: Involvement of the recurrent laryngeal nerve (RLN) in papillary thyroid carcinoma (PTC) is an important prognostic factor and is associated with a higher risk of recurrence. This study aimed to retrospectively analyze the outcomes of patients treated with hemithyroidectomy (HT) in PTC patients with an exclusive RLN invasion who could not tolerate staged surgery, did not wish to undergo another operation, or had other reasons. Methods: A retrospective review was conducted on 163 patients with PTC and exclusive RLN involvement at our institution between 2013 and 2019. Patients were divided into a total thyroidectomy (TT) group and HT group. The clinicopathologic factors and prognostic outcomes were compared between the two groups. A propensity score-matched analysis was carried out to reduce selection bias, with the following covariates: gender, age, tumor size, multifocality, central lymph node metastasis (CLNM), and RLN resection. The Kaplan–Meier method was used for a comparison of recurrence outcomes. Results: In the baseline data of the 163 PTC patients, tumor size (*p* < 0.001), multifocality (*p* = 0.011), CLNM (*p* < 0.001), and RLN resection (*p* < 0.008) in the TT and HT groups differed significantly, whereas age and gender did not differ between the two groups. The TT group reported significantly higher temporary and permanent hypoparathyroidism than the HT group (*p* < 0.001 and *p* = 0.042, respectively). With 72-month median follow-up, 11 (6.7%) patients developed recurrence. After propensity score matching, 24 patients with HT and 43 patients with TT were included. Recurrence-free survival (RFS) in the matched samples showed no difference between the TT and HT groups (*p* = 0.092). Conclusion: Our results indicate that HT may be a feasible treatment for PTC patients with exclusive RLN involvement in specific circumstances without significantly increasing the risk of recurrence. Performing a thorough preoperative examination is crucial to exclude multifocal tumors and lymph node metastasis before undergoing HT.

## 1. Introduction

Differentiated thyroid carcinoma (DTC) accounts for over 90% of all thyroid cancers and has a favorable prognosis with low mortality rates [1,2]. PTC represents the most frequent histological subtype of all thyroid malignancies and has excellent prognosis, as manifested by 10-year disease-specific survival (DSS) of approximately 96% [3]. Extrathyroidal extension (ETE) outside the thyroid capsule occurs in approximately 13% to 16% of patients and has important prognostic significance [4,5]. The presence of ETE is associated with an increased risk of persistent disease and recurrence [6]. The RLN was the most commonly infiltrated structure in case of ETE [7]. Approximately 33% to 61% of locally invasive thyroid cancers demonstrate RLN invasion [8,9,10].

Within the eighth edition of the American Joint Committee on Cancer/tumor node metastasis (AJCC/TNM) Staging System, the invasion of RLN was classified as T4a, regardless of tumor size [11]. The RLN invasion appears to be associated with an increased risk of recurrence in patients with PTC and represents a poor prognostic factor. This represents a poor prognostic factor for these patients. According to the American Thyroid Association (ATA) guidelines, the risk of recurrence in patients with gross extrathyroidal extension ranges from 23% to 40% [12]. In a previous study, tumor recurrence occurred 3 times more frequently in patients with an RLN invasion than patients without an invasion [13]. At the same time, the RLN invasion is often accompanied by an invasion of surrounding vital organs, such as major vessels, larynx, esophagus, and trachea, which increases the range of surgery and worsens the prognosis [14]. Thus, the poorer prognosis of PTC patients with RLN involvement may be related to the invasion of the peripheral structures. Some studies have revealed that compared to other locally invasive structures, the involvement of RLN had no significant influence on survival [10,15].

Hence, resecting an invaded RLN did not offer a statistically significant benefit regarding 5-year RFS [16]. Resection of tumor-involved RLN was recommended if preoperative vocal cord laryngoscopy and a voice examination were used to identify non-functional RLN [17]. When vocal cord function is normal preoperatively and the tumor is minimally adherent to RLN, shaving the primary tumor from the involved RLN is recommended [4,15,18]. For PTC patients with exclusive tumor involvement of the RLN, surgical approaches have become conservative. On the other hand, PTC patients with RLN involvement were difficult to find during preoperative assessments. Preoperative vocal cord paralysis occurred in approximately 35–70% of patients with an RLN invasion [19,20]. Performing contralateral thyroidectomy without intraoperative nerve monitoring (IONM) may be challenging when RLN involvement is found during surgery. It may result in bilateral RLN injury, which could require a tracheostomy for the patient. The use of staged surgical removal of the contralateral thyroid gland may be safer [21,22]. However, it has not yet been studied whether HT is suitable for PTC patients with an exclusive RLN invasion who cannot tolerate staged surgery, do not wish to undergo another operation, or have other reasons. Therefore, in the present study, we aimed to assess the outcomes of patients with an RLN involvement treated with HT under specific conditions.

## 2. Patients and Methods

A retrospective review was conducted of 163 PTC patients who had an exclusive local invasion of the RLN between December 2013 and December 2019 in the Department of Thyroid Surgery of the First Affiliated Hospital, School of Medicine, Zhejiang University. All patients underwent a preoperative ultrasonography evaluation of thyroid nodules and cervical lymph nodes. Each patient underwent an ultrasound-guided fine-needle aspiration biopsy (FNAB) for the diagnosis of thyroid gland malignancy and central or lateral compartment lymph nodes suspicious for metastasis. Computed tomography scans were performed in conjunction with ultrasound as important preoperative evaluations that could indicate the presence of an RLN invasion. A preoperative laryngoscopic assessment of the vocal cords was routinely performed.

All patients included in this study underwent thyroidectomy, including TT + bilateral central lymph node dissection (CLND) (level VI and level VII), or lobectomy with isthmectomy + ipsilateral CLND by the same group of surgeons using a standardized technique. The therapeutic strategy for invasive PTC was maximum resection of the tumor while maximally preserving the function of the RLN. Nerve preservation was attempted to avoid nerve injury when it was possible to isolate the RLN from the tumor through a sharp dissection. However, when the RLN was paralyzed and could not be easily shaved off the tumors, complete resection and functional reconstruction were performed for involved RLN. The reconstruction included anastomosis between the two segments of the RLN or between the RLN and the ansa cervicalis. The 129 patients who underwent TT received high-dose (100–150 mCi) RAI therapy 2 to 3 months after surgery.

The inclusion criteria were as follows: (1) patients who were pathologically diagnosed with PTC, (2) patients who underwent thyroid surgical treatment and with an RLN invasion due to thyroid carcinoma, and (3) patients did not have a history of thyroid surgery or a history of neck radiotherapy. Patients with a concurrent involvement of other structures were excluded from this study. One hundred and sixty-nine PTC patients with a local invasion of the RLN without an involvement of adjacent structures were enrolled. Of these patients, 6 were excluded because of distant metastasis (*n* = 1), bilateral RLN involvement (*n* = 2), previous thyroid surgery (*n* = 2), and previous neck radiation therapy (*n* = 1). Patients were assigned to two groups based on the surgical approach: the HT (lobectomy + isthmectomy) group and the TT group. This study was conducted in accordance with the Declaration of Helsinki and was approved by the Clinical Research Ethics Committee of the First Affiliated Hospital, School of Medicine, Zhejiang University (Ethic Code: IIT20240533A). All patients provided written informed consent before this study began.

Follow-up visits were performed every 3 months for the first 2 years and every 6 months for the following years. The mean follow-up was 68.9 ± 21.4 months (median, 72 months). All patients received thyroid-stimulating hormone (TSH) suppressive therapy. FNAB and cervical computerized tomography were used in the follow-ups to assess potential recurrences. Reoperation was performed on patients suspected of recurrence, and postoperative pathology was confirmed.

## 3. Statistical Analysis

Statistical analysis was performed using SPSS version 25 (SPSS, Inc., Chicago, IL, USA) and R software version (3.6.1). The results were expressed as mean ± SD. Differences between categorical variables were assessed using the Pearson χ^2^ test or Fisher exact test, and continuous variables were assessed using Student’s *t*-test. Univariable and multivariable Cox proportional hazards models were used to analyze the relationship between clinicopathological variables and RFS in the whole cohort. RFS rates were analyzed between the matched patients using Kaplan–Meier statistics and log-rank tests. Results were reported as hazard ratios (HRs) and 95% confidence intervals (95% CIs). A two-sided *p* value of <0.05 was defined as statistically significant.

Propensity score matching (PSM) was used to effectively minimize the selection bias and confounding differences. The model was adjusted for the following six variables: gender, age, tumor size, multifocality, central lymph node metastasis, and RLN resection. Before matching, the mean propensity score was 0.471 for patients in the HT group and 0.139 for patients in the TT group. To optimize the precision of this study, patients with HT were matched to patients with TT in a 1:3 matching ratio. The majority of patients with HT were successfully matched to three patients with TT, although some were matched to fewer patients, depending on their scores. A total of 67 patients were compared using the nearest neighbors method. After matching, the mean propensity score was 0.370 for patients in the HT group (*n* = 24) and 0.370 for patients in the TT group (*n* = 43).

## 4. Results

### 4.1. Baseline Characteristics of the Patient

A total of 163 PTC patients who had an exclusive local invasion to the RLN (128 women and 35 men) were analyzed retrospectively. In total, 34 patients underwent HT + ipsilateral CLND and 129 patients underwent TT + bilateral CLND. Among the 129 patients who underwent TT, 33 patients underwent a lateral lymph node dissection (levels IIa to Vb) simultaneously. Out of the 34 patients in the HT group, 6 were unable to tolerate the longer operation time and postoperative complications due to their older age or comorbidities. Fourteen patients with a potential preoperative RLN invasion opted for HT and a tight postoperative follow-up, after providing informed consent. Four patients with a tumor size less than 5 mm and five patients with a minimal invasion underwent HT. In the remaining five patients, the reason for HT was unclear.

The male-to-female ratio was 1:3.66, with a mean age of 46.36 ± 13.15 years (range, 13–86) at the time of surgery. The average size of the tumors was 1.43 ± 0.86 cm, and 43.6% of tumors were ≤1 cm in size. After the RLN invasion was intraoperatively confirmed, 141 (86.5%) patients underwent nerve preservation by shaving the primary tumor from the involved RLN, while the remaining 22 RLN (13.5%) patients underwent nerve resection. Moreover, 93/163 (57.1%) patients were defined as histologically positive for CLNM after surgery. Further, 33/163 (20.2%) patients underwent lateral lymph node dissection and 30/33 (90.9%) exhibited lateral lymph node metastasis. Multifocal tumors were found in 65 (39.9%) patients. Table 1 provides a summary of the baseline characteristics.

### 4.2. Propensity Score Matching and Analysis

There was no difference in the baseline characteristics between the two groups with respect to age, gender, thyroglobulin, thyroidperoxidase antibodies, thyroid-stimulating hormone, and recurrence (*p* = 0.842, *p* = 0.643, *p* = 0.209, *p* = 0.717, *p* = 0.335 and *p* = 0.588, respectively). In comparison to the HT group, the TT group had a considerably higher percentage of tumors > 1 cm (66.7% vs. 17.6%, *p* < 0.001). Multifocality, CLNM, and RLN resection were more frequent in the TT group than in the HT group, and this difference was statistically significant (*p* = 0.011, *p* < 0.001 and *p* = 0.008, respectively). The patients in the TT group had comparatively more metastases and dissections of the central lymph node (*p* = 0.008 and *p* = 0.043, respectively). No patients underwent an RLN resection in the HT group, while 22 patients (17.1%) underwent an RLN resection in the TT group. PSM was performed to generate matched groups. Overall, 24 patients from 34 patients who received HT and 43 patients from 129 patients who received TT were matched. The clinicopathological characteristics after PSM are presented in Table 2. There was no significant difference in the clinicopathologic characteristics between the two groups after matching.

### 4.3. Clinicopathologic Characteristics’ Comparison of Patients with ≤1 cm PTC 

About 82.4% of the patients with HT had tumors ≤ 1 cm, and 2/3 of the patients with TT had tumors > 1 cm. A subgroup analysis of the clinicopathologic characteristics of the patients with PTC ≤ 1 cm is shown in Table 3. The rates of multifocal tumors (5/28 [17.9%] vs. 21/3 [48.8%]; *p* = 0.007) and CLNMs (7/28 [25.0%] vs. 27/43 [62.8%]; *p* = 0.002) were significantly lower in the HT group than in the TT group. The two groups had comparable numbers of CLNDs. However, the mean number of lymph node metastases was significantly less in the HT group than in the TT group (0.75 ± 1.90 vs. 2.05 ± 2.71, *p* = 0.031). There were no statistically significant differences in age, gender, tumor size, rate of RLN resection, and recurrence (*p* = 0.362, *p* = 0.598, *p* = 0.359, *p* = 0.273 and *p* = 0.656, respectively).

### 4.4. Postoperative Tumor Recurrence

**Figure 1:**
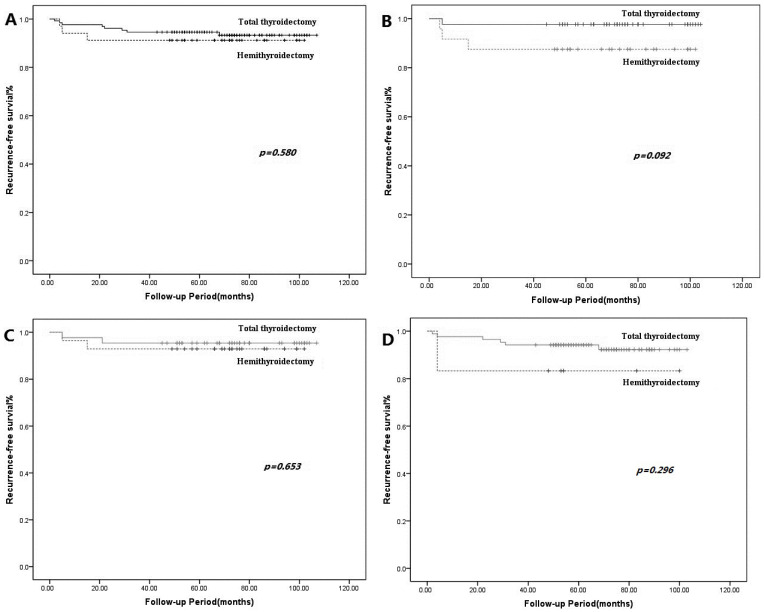
Recurrence-free survival curves in the total thyroidectomy and hemithyroidectomy groups before (**A**) and after (**B**) propensity score matching. Recurrence-free survival curves between total thyroidectomy and hemithyroidectomy groups in ≤1 cm (**C**) and >1 cm (**D**) subgroups. Kaplan–Meier method for recurrence with the log-rank test was used for statistical comparisons.

During the follow-up period, 11 (6.75%) patients developed recurrence. The mean time to recurrence after surgery was 18.7 ± 19.5 months. When comparing the clinicopathological variables of the 11 patients with recurrence to those of the 152 patients without recurrence, no statistically significant differences were found between the two groups (Table 4). Kaplan–Meier survival analysis with the log-rank test was performed to predict prognosis. The entire cohort’s 5-year RFS was 92.9%. The 5-year RFS of the TT group and HT group was 94.6% and 91.2%, respectively. Between the TT and HT groups, there was no significant difference before (Figure 1A) and after (Figure 1B) PSM (*p* = 0.580 and *p* = 0.092, respectively). In the matched group, recurrence occurred in 1 (2.3%) case within the TT group and 3 (12.5%) cases within the HT group. There were no statistically significant differences in recurrence between the two groups in the ≤1 cm and >1 cm subgroups (*p* = 0.653 and *p* = 0.296, respectively) (Figure 1C,D).

Among the recurrent patients, 9 (81.8%) patients had metastases in the lateral neck compartment, 1 (9.1%) patient had metastases in the ipsilateral central lymph nodes, and 1 (9.1%) patient had metastases in the contralateral thyroid gland. None of the patients experienced local recurrence at the site of the RLN invasion. Table 5 summarizes the 11 patients who experienced tumor recurrence. After surgery, the recurrence time ranges from 2 to 68 months. Of these recurrence patients, the rate of CLNM and multifocality were 54.5% and 36.4%, respectively. Two patients developed bilateral lateral lymph node metastases. Re-operative surgery was performed in patients with tumor recurrence.

### 4.5. Postoperative Complications

In the HT group, vocal fold palsy was confirmed in 27 patients (79.4%) on the side of HT. In contrast, 107 patients (82.9%) were documented in the TT group (*p* = 0.632). Meanwhile, the incidence of temporary and permanent (more than 12 months) hypoparathyroidism was significantly lower in HT group compared to the TT group (temporary hypoparathyroidism: 17.6% vs. 72.1%, respectively, *p* < 0.001; permanent hypoparathyroidism: 0% vs. 10.1%, respectively, *p* = 0.042). None of the patients in either group had a hematoma (Table 6).

## 5. Discussion

Although DTC patients generally have an excellent prognosis with a survival rate up to 98% [23,24], some patients may experience local recurrence or distant metastasis due to clinical features with high aggressiveness [25]. Previous studies have reported the 10-year recurrence rate of DTC as 21–25% [26,27]. The risk factors influencing recurrence include patients aged ≥55 years, extrathyroid extension, multifocality, lymph nodal and distant metastases, tumor size > 2 cm, aggressive histologic subtypes, etc. [28,29]. The presence of ETE occurs in up to 30% of DTC and has been recognized as a potential predictor of worse outcomes [12,30]. In the 8th version of the AJCC staging system published in 2018, ETE has been linked to the TNM stage and influences the selection of treatment options [14]. In the study of Clain et al., patients with ETE were 12 times more likely to have lymph nodes metastases than patients with intrathyroidal primary tumors [31]. Michael D et al. have demonstrated that ETE was associated with an increased risk for distant metastasis [32]. For these reasons, the ATA guidelines recommend TT for patients with gross ETE [33].

In the AJCC staging system, macroscopic ETE of subcutaneous soft tissue, larynx, trachea, major vessels, esophagus, or RLN is classified as T4a, regardless of tumor size. The anatomic position of the RLN in relation to the thyroid gland puts it at risk of being affected by disease processes. Due to its unique anatomy, the RLN is the second most commonly invaded structure by primary or metastatic thyroid cancer [7,34]. Approximately 33% to 61% of locally invasive thyroid tumors demonstrate an RLN invasion [8,9,10,35]. The optimal surgical management of patients with an RLN invasion remains controversial. The importance of surgical completeness of locally invasive PTC has been emphasized by previous studies [5]. Some surgeons have expressed concern that shaving extensive tumors may result in microscopically positive margins [36]. However, PTC with an exclusive RLN involvement treated by nerve shaving or resection has not significantly influenced survival [4,15,18,37,38]. Falk et al. demonstrated that complete excision of PTC with resection of the RLN did not improve survival compared to incomplete excision [39]. In the study of Yang et al., tumor shaving showed superiority in preserving RLN function without increasing the risk of recurrence in patients with papillary thyroid microcarcinoma [40]. The RLN invasion was commonly associated with other invaded structures. Comparing locally invasive PTC to other structures, exclusive involvement of the RLN may be a distinct situation. The poorer prognosis of PTC patients with an RLN invasion indicated in previous studies may be related to the invasion of peripheral structures.

Ideally, initial treatment should offer the best disease control and survival outcomes without compromising safety concerns. Compared to HT, TT may be a better option for preventing disease recurrence. However, even though it is a predictive factor for disease-specific recurrence, a bilateral RLN injury with corresponding irreversible tracheostomy is an undesirable outcome for the patient. The criteria for determining preoperative RLN involvement have been limited in previous studies. Neither ultrasonography nor other cross-sectional imaging can reliably identify the entire course of the RLN. The presence of RLN invasions may be assessed in patients with hoarseness or by laryngoscopy (to observe vocal cord movement). There were still patients experiencing hoarseness and decreased vocal cord movement without an invasion of the RLN [16]. This may be due to local inflammation of the RLN caused by tumor compression. In contrast, Kamani et al. reported that 50% of invaded RLNs presented with normal preoperative laryngeal function [41]. When the RLN is unexpectedly found to be invaded by a tumor during surgery, and the nerve has to be resected or be shaved without guaranteeing its function; resection of the contralateral thyroid gland may be challenging without the use of IONM. Staged surgery was sometimes performed to reduce the risk of a bilateral RLN injury and airway compromise when there was signal loss on the first surgical side [42], which may be an effective strategy to improve patients’ quality of life [21].

However, for PTC patients with an exclusive RLN invasion due to a primary thyroid tumor without an involvement of adjacent structures, it has not been studied whether HT is appropriate. RFS is an important survival outcome for deciding the extent of surgery. In total, 20 (40%) patients with an RLN invasion in the study of Toshirou et al. underwent HT. However, the prognosis of these patients and the difference in clinical characteristics from patients with TT were not mentioned [10]. In the study by Brooks et al., 28 patients underwent HT, and 17 of them underwent staged or completion surgery, but the prognosis of these patients was not stated [16]. In this study, 34 of 163 (20.9%) patients underwent HT. The recurrence rates for the HT and TT groups were 8.8% and 6.2%, respectively. Although the tumor size in the TT group was larger than that in the HT group, and the rates of multifocality and lymph node metastasis were significantly higher than those in the HT group, there was no significant difference in recurrence rates between the two groups. Meanwhile, compared with previously published results for all PTC patients from our institute, patients who underwent HT in this study had smaller tumor sizes, lower rates of lymph node metastasis, and lower multifocality [43]. In addition, the rate of RLN resection in the TT group was 17.1%, which was higher than that in the HT group (0%). This means that although there were no clear criteria for patients with HT in this study, clinical characteristics with a low recurrence risk were favored in the selection of patients.

For PTC patients who cannot tolerate staged surgery, do not want to undergo another surgery, or for other reasons, HT alone may be an appropriate surgical procedure. Therefore, the aim of the present study was to evaluate the outcome of patients with an exclusive RLN involvement treated with HT under specific conditions. Our study used the PSM method to balance the bias which could be generated from gender, tumor size, age, multifocality, central lymph node metastasis, and RLN resection. Thus, the selection bias could be ignored when different types of surgery were compared. The recurrence rates for the HT and TT groups after matching were 12.5% and 2.3%, respectively. Matching and weighting are the most common methods in utilizing PSM. This process may have increased the risk factors for the HT group or decreased the risk factors for the TT group, ultimately achieving a match between the two groups. Although the TT group had a lower recurrence rate than the HT group during follow-up, the recurrence rate was not statistically different. On the other hand, previous studies observed limited hemithyroidectomy in relation to papillary thyroid microcarcinoma. Papillary thyroid microcarcinoma in the TT group still had high rates of multifocality and central lymph node metastasis in this study. However, the three patients who relapsed in the HT group did not experience a recurrence in level VI lymph nodes. One patient developed contralateral thyroid recurrence, in which no nodule existed on the preoperative ultrasound. Therefore, patients were carefully selected for HT following specific criteria: no suspicious nodule on the contralateral thyroid gland and no suspicious cervical lymph nodes either on the preoperative ultrasound or on the intraoperative palpation.

The other potential advantage of HT is the lower incidence of complications. A meta-analysis showed significant differences of hypoparathyroidism and RLN injury for patients who received TT compared with HT [44]. According to a recent study, the median incidence of temporary and permanent hypoparathyroidism following TT ranges from 19–38% and 0–3%, respectively [45,46]. The present study revealed that the TT group had a higher rate of temporary and permanent hypoparathyroidism than the HT group (72.1% vs. 17.6%, *p* < 0.001 and 10.1% vs. 0%, *p* = 0.042, respectively). The incidence of transient and permanent hypoparathyroidism in patients who underwent total thyroidectomy in this study was higher than in the previous studies. This may be related to the complex surgical environment and central lymph node dissection. In the TT group, 65.1% of patients had CLNM. Previous studies have indicated that a central lymph node dissection was a risk factor for hypoparathyroidism [47]. The complete removal of the central lymph nodes may result in a reduction in the blood supply to the parathyroid glands, which could potentially lead to hypoparathyroidism. The rates of postoperative hoarseness and hematoma did not differ between the two groups.

Our study is not without its limitations. First, despite the PSM being used in our study, its retrospective form means that it cannot completely replace the randomization procedure. Meanwhile, the PSM was produced using available covariates. However, this could not eliminate the potential bias caused by unmeasured variables. Multicenter randomized clinical trials in the future could improve the level of scientific evidence of these conclusions. Second, given that the incidence of PTC with an exclusive RLN involvement was low, selection bias may have been present due to the small number of patients in this research study. TT was commonly chosen for PTC with an RLN involvement in our institute; the number of patients that underwent HT was restricted. Finally, the relatively short follow-up period may not have been sufficient to identify all recurrences. To address the limitations of this study, more prospective studies with longer follow-up times and a larger sample size are required.

## 6. Conclusions

In summary, our results indicate that HT does not increase the risk of postoperative recurrence for PTC patients with an exclusive RLN involvement who cannot tolerate prolonged surgery, do not wish to undergo staged surgery, and without the use of IONM instruments. HT may be a feasible surgical procedure for PTC patients with an exclusive RLN involvement in specific circumstances. A thorough preoperative examination is crucial to exclude multifocal tumors and lymph node metastases before undergoing HT.

## Figures and Tables

**Table 1:** Clinical and pathological characteristics of PTC patients with exclusive RLN invasion.

Patient Characteristics	Total (*n* = 163)
Sex	
Male, *n* (%)	35 (21.5%)
Female, *n* (%)	128 (78.5%)
Age, years, mean ± SD (range)	46.36 ± 13.15 (13–86)
RLN invasion side, *n* (%)	
Right	91 (55.83%)
Left	70 (42.94%)
Bilateral	2 (1.23%)
RLN resected, *n* (%)	
Yes	22 (13.5%)
No	141 (86.5%)
Tumor size, cm, mean ± SD	1.43 ± 0.86
≤1 cm, *n* (%)	71 (43.6%)
>1 cm, *n* (%)	92 (56.4%)
CLNM, *n* (%)	
Yes	93 (57.1%)
No	70 (42.9%)
Lateral neck metastases, *n* (%)	
Yes	30/33 (90.9%)
No	3/33 (9.1%)
Multifocality, *n* (%)	
Yes	65 (39.9%)
No	98 (60.1%)
Recurrence, *n* (%)	
Yes	11 (6.75%)
No	152 (93.25%)
TG, ng/mL (mean ± SD)	74.04 ± 262.89
TPOAb, IU/mL (mean ± SD)	461.20 ± 1321.12
TSH, µIU/mL (mean ± SD)	2.46 ± 3.61
Follow-up time, years, median (range)	72 (2–107)

RLN, recurrent laryngeal nerve; CLNM, central lymph node metastasis; TG, thyroglobulin; TPOAb, thyroid peroxidase antibody; TSH, thyroid-stimulating hormone.

**Table 2:** Comparison of clinicopathologic characteristics of patients with RLN involvements between hemithyroidectomy and total thyroidectomy before and after propensity score matching.

Characteristics	Before Matching (*n* = 163)		*p* Value	After Matching (*n* = 67)		*p* Value
	Hemithyroidectomy (*n* = 34)	Total Thyroidectomy (*n* = 129)		Hemithyroidectomy (*n* = 24)	Total Thyroidectomy (*n* = 43)	
Age (mean ± SD, years)	46.8 ± 13.38	46.3 ± 13.13	0.842	46.3 ± 13.74	49.9 ± 13.63	0.306
<55	22 (64.7%)	99 (76.7%)	0.186	16 (66.7%)	28 (65.1%)	0.898
≥55	12 (35.3%)	30 (23.3%)		8 (33.3%)	15 (34.9%)	
No. of females, *n* (%)	28 (82.4%)	100 (77.5%)	0.643	21 (87.5%)	35 (81.4%)	0.518
Size of tumor, cm	0.88 ± 0.47	1.58 ± 0.88	<0.001	0.91 ± 0.55	0.97 ± 0.47	0.655
≤1 cm	28 (82.4%)	43 (33.3%)	<0.001	18 (75.0%)	30 (69.8%)	0.649
>1 cm	6 (17.6%)	86 (66.7%)		6 (25.0%)	13 (30.2%)	
Multifocality, *n* (%)	7 (20.6%)	58 (45.0%)	0.011	7 (29.2%)	15 (34.9%)	0.633
CLNM, *n* (%)	9 (26.5%)	84 (65.1%)	<0.001	9 (37.5%)	21 (48.8%)	0.371
Mean no. dissected nodes for CND	6.56 ± 3.32	8.50 ± 5.28	0.043	6.58 ± 3.73	8.16 ± 5.59	0.221
Mean no. metastasis nodes for CND	0.91 ± 2.08	2.63 ± 3.43	0.006	1.29 ± 2.39	1.88 ± 3.33	0.446
RLN resection, *n* (%)	0 (0%)	22 (17.1%)	0.008	0 (0%)	0 (0%)	NA
TG (mean ± SD, ng/mL)	23.59 ± 34.85	87.34 ± 293.77	0.209	21.68 ± 37.45	52.07 ± 91.92	0.127
TPOAb (mean ± SD, IU/mL)	387.79 ± 761.36	480.56 ± 1434.48	0.717	409.56 ± 685.54	439.88 ± 1337.49	0.918
TSH (mean ± SD, µIU/mL)	3.00 ± 6.80	2.32 ± 2.12	0.335	3.54 ± 8.07	2.57 ± 2.49	0.470
Recurrence, *n* (%)	3 (8.8%)	8 (6.2%)	0.588	3 (12.5%)	1 (2.3%)	0.092

RLN, recurrent laryngeal nerve; CLNM, central lymph node metastasis; CND, central neck dissection; TG, thyroglobulin; TPOAb, thyroid peroxidase antibody; TSH, thyroid-stimulating hormone.

**Table 3:** Comparison of clinicopathologic characteristics of ≤1 cm PTC patients with RLN involvements between hemithyroidectomy and total thyroidectomy groups.

Characteristics	Hemithyroidectomy (*n* = 28)	Total Thyroidectomy (*n* = 43)	*p* Value
Age (mean ± SD, years)	46.04 ± 12.27	48.67 ± 11.56	0.362
<55	20 (71.4%)	31 (72.1%)	0.579
≥55	8 (28.6%)	12 (27.9%)	
No. of females, *n* (%)	23 (82.1%)	35 (81.4%)	0.598
Size of tumor, cm	0.72 ± 0.21	0.76 ± 0.20	0.359
Multifocality, *n* (%)	5 (17.9%)	21 (48.8%)	0.007
CLNM, *n* (%)	7 (25.0%)	27 (62.8%)	0.002
Mean no. dissected nodes for CND	6.79 ± 3.19	8.30 ± 4.86	0.149
Mean no. metastasis nodes for CND	0.75 ± 1.90	2.05 ± 2.71	0.031
RLN resection, *n* (%)	0 (0%)	3 (7.0%)	0.273
TG (mean ± SD, ng/mL)	22.29 ± 35.72	52.02 ± 86.93	0.091
TPOAb (mean ± SD, IU/mL)	431.66 ± 818.82	402.96 ± 1378.80	0.921
TSH (mean ± SD, µIU/mL)	3.30 ± 7.47	2.77 ± 3.15	0.680
Recurrence, *n* (%)	2 (7.1%)	2 (4.7%)	0.656

RLN, recurrent laryngeal nerve; CLNM, central lymph node metastasis; CND, central neck dissection; TG, thyroglobulin; TPOAb, thyroid peroxidase antibody; TSH, thyroid-stimulating hormone.

**Table 4:** Clinicopathologic factors related to tumor recurrence.

Characteristics	Recurrence (*n* = 11)	No Recurrence (*n* = 152)	*p* Value
Age (years), mean ± SD	45.64 ± 11.59	46.41 ± 13.29	0.850
Age < 55 years, *n* (%)	8 (72.7%)	113 (74.3%)	0.906
Tumor size ≤ 1 cm, *n* (%)	4 (36.4%)	67 (44.1%)	0.758
Female, *n* (%)	11 (100%)	117 (77.0%)	0.123
CLNM, *n* (%)	6 (54.5%)	87 (57.2%)	0.862
Mean no. dissected nodes for CND	7.55 ± 5.56	8.14 ± 4.96	0.705
Mean no. metastasis nodes for CND	1.55 ± 2.02	2.32 ± 3.34	0.448
Multifocality, *n* (%)	4 (36.4%)	61 (40.1%)	0.805
RLN resection	1 (9.1%)	21 (13.8%)	0.547
Hemithyroidectomy, *n* (%)	3 (27.3%)	31 (20.4%)	0.588

RLN, recurrent laryngeal nerve; CLNM, central lymph node metastasis; CND, central neck dissection.

**Table 5:** Characteristics of the patients with tumor recurrence.

Patient # No.	Surgery	Age, y	Sex	Size, mm	CLNM(%)	Multifocality	Time to Recurrence, m	Recurrence Site
1	HT	26	F	10	44.4%	-	5	Level III, IV lymph node
2	HT	42	F	10	0.0%	-	15	Contralateral thyroid gland
3	HT	58	F	15	0.0%	-	4	Level III, IV lymph node
4	TT	30	F	15	60.0%	-	4	Level IV lymph node
5	TT	34	F	18	27.3%	+	2	Bilateral level II, III, IV lymph node
6	TT	41	F	22	16.7%	-	68	Level II, III, IV lymph node
7	TT	52	F	15	50.0%	-	31	Level IV, V lymph node
8	TT	53	F	7	25.0%	-	21	Level VI lymph node
9	TT	53	F	20	0.0%	+	29	Bilateral level II, III, IV lymph node
10	TT	55	F	12	0.0%	+	22	Level II, III, IV lymph node
11	TT	58	F	7	0.0%	+	5	Level III, IV lymph node

HT, hemithyroidectomy; TT, total thyroidectomy; CLNM, central lymph node metastases. + represents multifocal tumors; - represents unifocal tumor.

**Table 6:** Adverse events between hemithyroidectomy and total thyroidectomy.

Characteristics	Hemithyroidectomy (*n* = 34)	Total Thyroidectomy (*n* = 129)	*p* Value
Hypoparathyroidism			
Temporary	6 (17.6%)	93 (72.1%)	<0.001
Permanent	0 (0%)	13 (10.1%)	0.042
Hoarseness	27 (79.4%)	107 (82.9%)	0.632
Hematoma	0 (0%)	0 (0%)	NA

NA, not available.

## Data Availability

The data presented in this study are available upon request from the corresponding author.
